# The BTB-Containing Protein Kctd15 Is SUMOylated *In Vivo*

**DOI:** 10.1371/journal.pone.0075016

**Published:** 2013-09-24

**Authors:** Valeria E. Zarelli, Igor B. Dawid

**Affiliations:** Program in Genomics of Differentiation, Eunice Kennedy Shriver National Institute of Child Health and Human Development, National Institutes of Health, Bethesda, Maryland, United States of America; Texas A&M University, United States of America

## Abstract

Potassium Channel Tetramerization Domain containing 15 (Kctd15) has a role in regulating the neural crest (NC) domain in the embryo. Kctd15 inhibits NC induction by antagonizing Wnt signaling and by interaction with the transcription factor AP-2α activation domain blocking its activity. Here we demonstrate that Kctd15 is SUMOylated by SUMO1 and SUMO2/3. Kctd15 contains a classical SUMO interacting motif, ψKxE, at the C-terminal end, and variants of the motif within the molecule. Kctd15 SUMOylation occurs exclusively in the C-terminal motif. Inability to be SUMOylated did not affect Kctd15's subcellular localization, or its ability to repress AP-2 transcriptional activity and to inhibit NC formation in zebrafish embryos. In contrast, a fusion of Kctd15 and SUMO had little effectiveness in AP-2 inhibition and in blocking of NC formation. These data suggest that the non-SUMOylated form of Kctd15 functions in NC development.

## Introduction

SUMOylation is a postranslational modification where the Small Ubiquitin-like Modifier (SUMO) is covalently attached to a target protein [1], [2]. SUMO conjugation of proteins that are involved in transcriptional regulation mediates control of gene expression [3], [4]. Often, this role is linked to repressive behavior. In vertebrates, four SUMO proteins are expressed: SUMO1, SUMO2, SUMO3 and SUMO4. Sequence homology clusters SUMO2 and 3 in the same subfamily, differing substantially from SUMO1, while SUMO4 has approximately 86% homology to SUMO2/3 and has a role in stress response [5], [6]. SUMO is attached to a Lysine contained in a tetrapeptide motif with the consensus ψ-K-x-E (ψ: a hydrophobic residue, K: lysine and E: an acidic residue) [7], [8], [9]. Some variations of the consensus site are SUMOylated in various proteins [10], [11]. An enzymatic cascade regulates protein modification by SUMO through a cycle of conjugation and deSUMOylation [12]. Substrate specificity is derived primarily from the SUMO-conjugating E2 enzyme UBC9, the motif in the substrate, and in some instances PIAS family E3 enzymes [13]. SENP1 isopeptidases are involved in the removal of SUMO from modified proteins [1], [14].

Kctd15 belongs to a family of proteins, the Potassium Channel Tetramerization Domain family, which are not channel proteins but are related because all harbor a BTB domain close to the N terminus. The function of Kctd proteins is still being characterized [15]. We have reported that Kctd15 has a role as an antagonist of neural crest (NC) formation [16], while other family members are implicated as adaptors for Cullin 3 ubiquitin-ligase [17], [18]. More recently, we have shown that Kctd15 strongly inhibits transcription factor AP-2α activity, explaining at least in part its impairment of NC development [19]. All Kctd proteins harbor a BTB domain that acts as a protein-protein interacting interface [20]. Whereas several BTB containing proteins contain additional functional domains such as Back or MATH domains [21], Kctd15 lacks a second recognizable domain. The activity of many proteins is regulated by posttranslational modification, and we considered the possibility of Kctd15 SUMOylation primarily because of its activity as a transcriptional inhibitor of AP-2. When the Kctd15 sequence was analyzed using the SUMOplot predictor program, we found a conserved high scoring SUMO interacting-motif (SIM) at the C-terminal end, in addition to other lower scoring motifs.

Here we demonstrate that the C-terminal recognition motif in Kctd15 is a target for SUMO1 and SUMO2/3 conjugation. Further, that a lysine (K) to arginine (R) mutation in this motif abolished SUMOylation, indicating that this is the only site in Kctd15 for SUMO modification. The non-SUMOylated form of Kctd15 showed the same subcellular localization and the same ability to suppress AP-2α activity and inhibit NC formation as the wild type protein.

## Results

### Kctd15 is a substrate for SUMOylation

**Figure 1:**
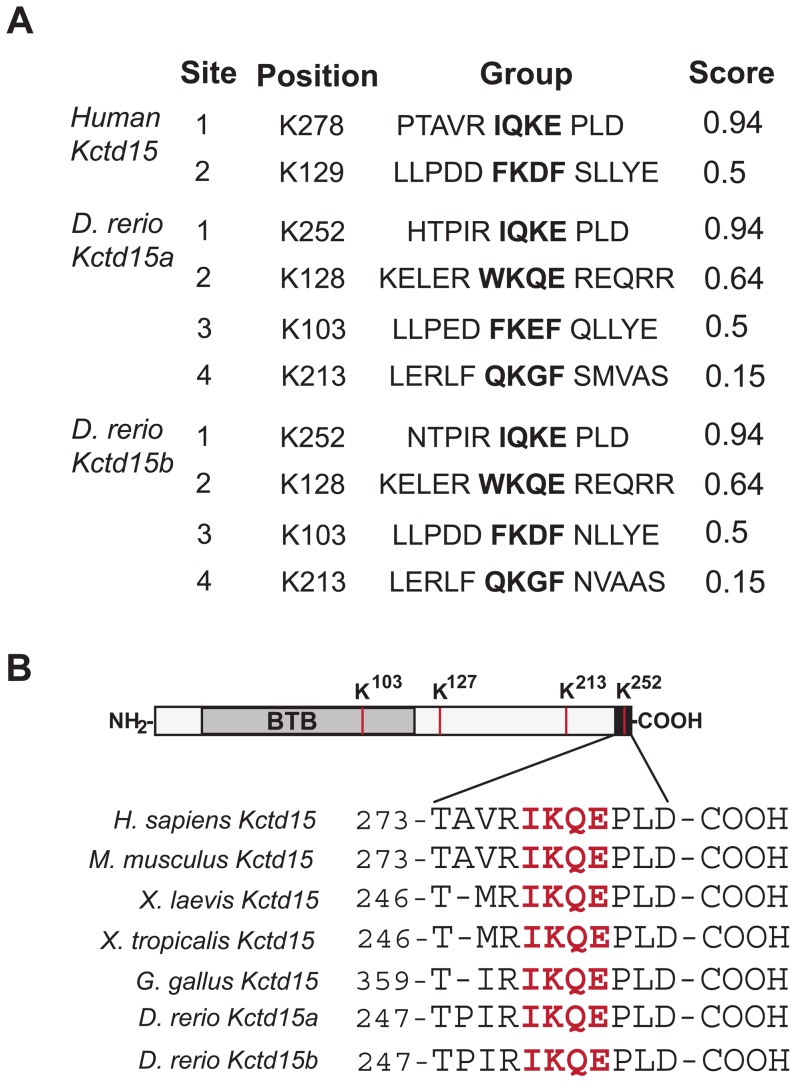
SUMOylation Motif in Kctd15. (A) SUMOylation sites in human KCTD15, and zebrafish Kctd15a and Kctd15b predicted by the SUMOplot Predictor Program, http://www.abgent.com/tools/. (B) Diagram of the BTB-containing protein zebrafish Kctd15. The different Lysine targets of SUMO are indicated in red. The C terminus of Kctd15 (black) harbors the highest confidence SUMO Interacting Motif (SIM). Sequence of the C-terminal region is shown with the SIM in red, demonstrating conservation between species.

**Figure 2:**
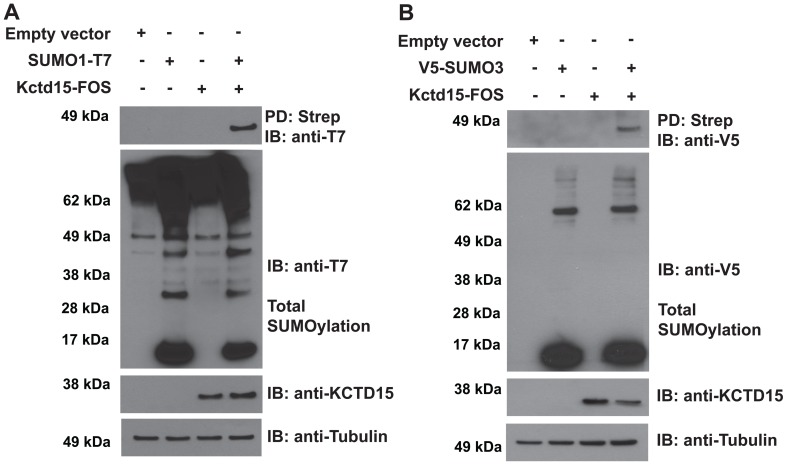
Kctd15 is a target for SUMOylation. (A) HEK293T cells were transfected with SUMO1-T7 with or without Kctd15-Flag-ONE-Strep (Kctd15-FOS). Cells were lysed in the presence of IAA and NEM to inhibit isopeptidase activity. Strep-Tactin pull down (PD) and immunoblotting (IB) are indicated; Tubulin was used as loading control. Immunoprecipitated Kctd15-FOS SUMOylated by SUMO1-T7 is observed in lane 4, upper panel, and total SUMOylation is shown in the second panel. (B) The same experimental approach as in (A), but using V5-SUMO3. Kctd15-FOS is SUMOylated by V5-SUMO3 (upper panel, lane 4).

Human and zebrafish Kctd15 sequences were analyzed by the SUMOplot Analysis Program (http://www.abgent.com/tools/) to predict SUMOylation sites. Due to duplication in the genome of teleost fish, two isoforms of Kctd15 occur in zebrafish, and homologous sites for SUMO conjugation were found in both paralogs (Figure 1A, 1B). In addition, all Kctd15 proteins of different species that were examined contain a well-conserved SIM in the C-terminal region (Figure 1B). We searched all known KCTD proteins for SIMs; many examples were found although the highest scoring SIMs are contained in Kctd15 and the closely related Kctd1 (Table S1). To determine whether Kctd15 is a target for SUMOylation, HEK293T cells were transfected with Kctd15-FOS [19] and T7 tagged SUMO1. Under regular cell lysis conditions SUMO is rapidly released from the target protein by endogenous isopeptidases. Thus, to preserve the SUMOylated form of Kctd15, we added isopeptidase inhibitors (IAA and NEM) to the lysis buffer, and carried out pull-down and blotting with anti-T7 antibody as described in Materials and Methods. A T7 epitope-positive band was detected at the expected molecular size of mono-SUMOylated Kctd15-FOS (Figure 2A). It is well known that SUMO1 attachment to a target lysine usually leads to mono-SUMOylated protein whereas SUMO2/3 can generate poly-SUMOylation by attachment to a lysine in SUMO [22], [23]. We next tested SUMO3 and found that Kctd15 was again mono-SUMOylated with no detectable poly-SUMOylation (Figure 2B). Because of the high homology between SUMO2 and 3 these two proteins are usually considered as equivalent in their behavior and identified as SUMO2/3.

### Kctd15 harbors a SUMO Interacting Motif at the C-terminal end

**Figure 3:**
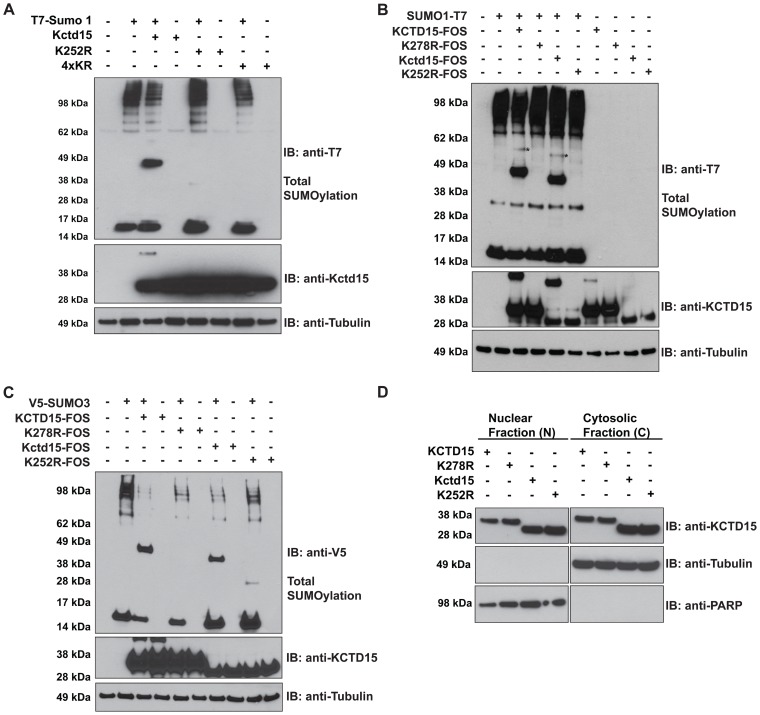
Identification of a SUMOylation site in Kctd15. (A) HEK293T cells expressing zebrafish WT Kctd15, K252R and 4xKR mutants in the presence or absence of SUMO1-T7 were lysed in hot SDS sample buffer (see Materials and Methods). SUMOylation was analyzed by using an anti-T7 antibody. A band of molecular size corresponding to the SUMOylated form of Kctd15 was observed in lane 3. The K252R and 4xKR mutants were not SUMOylated by SUMO1-T7 (lanes 5 and 7, respectively). The middle panel shows protein input; in lane 3 SUMOylated Kctd15 is detected. (B) In a similar way we checked SUMOylation of human KCTD15 and its mutant K278R, compared to zebrafish proteins. Upper panel lanes 3 and 5 show SUMOylation of both WT forms, while the middle panel shows protein inputs where SUMOylated forms of human and zebrafish Kctd15 are also detected. The band labeled by an asterisk does not correspond to any SUMOylated form of Kctd15 and is likely to represent background. Both K/R mutants are not SUMOylated. (C) SUMOylation of human and zebrafish Kctd15 was achieved by co-expression of V5-SUMO3 (lanes 3 and 7). Middle panel shows protein inputs detected with anti-Kctd15 antibody where is possible to see SUMOylated form of human KCTD15. (D) Subcellular fractionation of HEK293T cells overexpressing human or zebrafish Kctd15, and K278R or K252R mutants. An anti-Kctd15 antibody detected WT or mutant forms of Kctd15 in both compartments. Tubulin and PARP monitored cell fractionation. The bottom panels in (A), (B) and (C) show loading controls using anti-Tubulin antibody.

Mutation of all four potential SUMO acceptor sites in the Kctd15 molecule (4xKR) renders the protein deficient for SUMOylation (Figure 3A). Because all Kctd15 proteins studied harbor a well-conserved SIM close to the C terminus we mutated the relevant Lysine (278 in human and 252 in zebrafish) to arginine to generate KCTD15 (K278R) and Kctd15 (K252R), respectively. The wild type (WT) and mutant proteins were overexpressed in HEK293T cells and their ability to be SUMOylated was compared. In these experiments we prevented isopeptidase-mediated deSUMOylation by instantly dissolving the cells in hot SDS sample buffer [24]. While in the case of WT proteins a band was detected at the position expected for mono-SUMOylated Kctd15, K278R and K252R mutant proteins failed to generate this band, which was likewise the case with the 4xKR mutant (Figure 3A, 3B). Note that the SUMOylated form of Kctd15 that was detected in whole lysates by blotting with anti-Kctd15 or anti-SUMO antibodies, indicating that a rather high proportion of total Kctd15 protein was SUMOylated under these conditions. The same result was obtained when V5-SUMO3 was used to accomplish SUMOylation (Figure 3C). These results indicate that the major if not unique site capable of SUMO conjugation in Kctd15 involves the SIM close to the C terminus.

### SUMOylation does not affect the subcellular localization of Kctd15

It is known that SUMO modification of target proteins can alter their subcellular localization and, as a consequence, modify their function and interaction with their partners [25], [26], [27]. Previously, we have shown that Kctd15 is almost equally distributed between nuclear and cytosolic fractions [19]. To test if mutation in the SUMO acceptor site might affect Kctd15 localization, we transiently overexpressed WT and K/R mutant human and zebrafish Kctd15 in HEK293T cells, and isolated nuclear and cytoplasmic fractions 24 hours later. Equivalent amounts of both fractions were loaded on SDS-PAGE gels and stained with anti-Kctd15 antibody. We observed that the K278R and K252R mutants of human and zebrafish Kctd15 exhibited the same compartmentalization within the cell as the WT form (Figure 3D).

### SUMOylation of Kctd15 is not required to inhibit AP-2 activity or NC development

**Figure 4:**
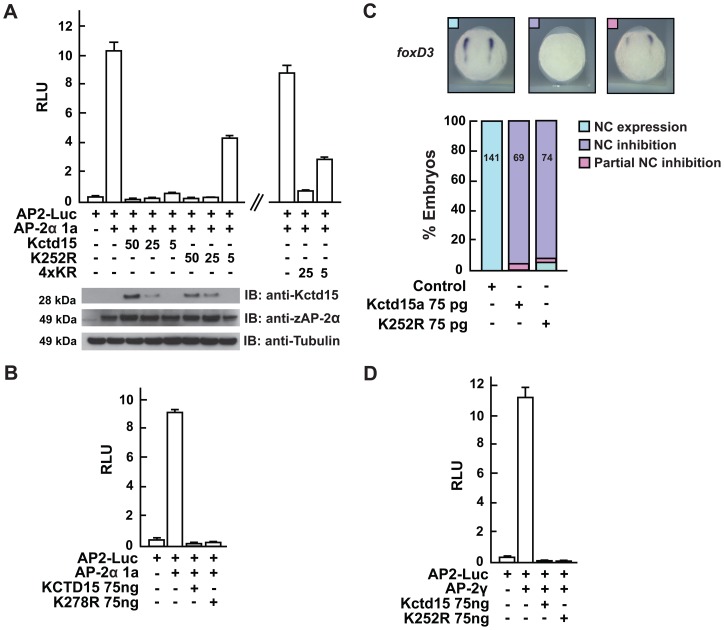
KR mutants repress AP-2α function. (A) AP-2α activity in a reporter assay [19] was inhibited by the K252R and 4xKR mutants with similar efficacy as the WT protein, although inhibition was reduced at low concentration. Immunoblots with anti-Kctd15, anti-AP-2 and anti-Tubulin antibodies control for protein expression. (B) AP2-Luc reporter assay using human KCTD15 and K278R mutant, both of which inhibit activity. (C) Kctd15 and K252R mRNAs were injected into zebrafish embryos. ISH with *foxD3* probe at the 1 somite stage shows that the K252R mutant inhibits NC formation as effectively as WT Kctd15. Embryo images define normal expression, total inhibition and partial inhibition. Quantification is shown in the histogram, numbers of embryos listed on the bars. (D) Luciferase assay using AP-2γ illustrates that zebrafish WT and K252R mutant are equally effective in inhibiting reporter activity.

Previous results from our laboratory have shown that Kctd15 regulates AP-2α activity through binding to the activation domain [19]. AP-2 family transcription factors are involved in several developmental processes, playing a major role in NC establishment [28], [29], [30]. We have demonstrated that interference with AP-2 activity is the basis, at least in part, for the inhibition of NC formation in zebrafish embryos by Kctd15 [19]. Because SUMO modification correlates with transcriptional repression [3], [4], [31], [32], [33] we pursued the hypothesis that SUMOylation of Kctd15 may be necessary for its inhibition of AP-2 activity. We therefore tested WT and K/R mutant Kctd15 in an AP-2 reporter assay. We expressed the AP2-Luc reporter with AP-2α and WT or K/R mutant Kctd15 in HEK293T cells, and measured luciferase activity. As shown in Figure 4A, zebrafish Kctd15 (4xKR) and (K252R) affected AP-2α activity as dramatically as WT Kctd15; human KCTD15 (K278R) showed similar behavior (Figure 4B). To examine whether this result depends on the cellular environment and properties of the reporter assay we injected WT and mutant Kctd15 into zebrafish embryos and tested for NC development. In situ hybridization with *foxD3* probe revealed that the K252R mutant inhibits NC formation as efficiently as WT Kctd15 (Figure 4C). These observations suggest that SUMO modification of Kctd15 is not involved in the ability of Kctd15 to block NC development. Three isoforms of AP-2 are expressed in zebrafish embryos, and AP-2α acts in concert with AP-2γ during NC formation [30]. Therefore we asked if Kctd15 (K252R) inhibits AP-2γ activity as it does AP-2α, and found that this is the case (Figure 4D).

### Kctd15-SUMO1-fusion protein is less effective as an inhibitor of AP-2 activity and NC development

**Figure 5:**
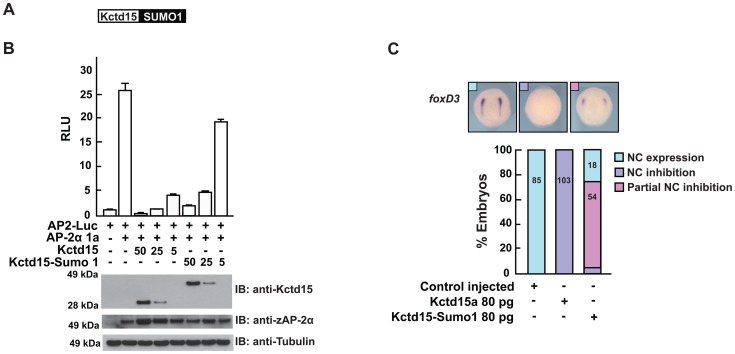
Kctd15 conjugated to SUMO1 is less competent in inhibiting AP-2α activity and NC formation. (A) Diagram of the fusion protein between Kctd15 and SUMO1. (B) Kctd15-SUMO1 conjugated protein was less effective than WT in repressing AP-2α dependent reporter activation. (C) Injection into zebrafish embryos of Kctd15-SUMO1 failed to abolish NC formation.

SUMO conjugation and deconjugation are dynamic processes. While much is known about the regulatory properties of SUMO attachment less attention has been devoted to the functional consequences of SUMO removal. Several lines of evidence suggest that deSUMOylation positively regulates protein activity; SUMO removal from potassium channel K2P1 allows channel activity [34], SUMO detachment from GATA1 favors its DNA binding and erythropoietic activity [35], deSUMOylation regulates polycomb repressor complex [36], and IFN regulatory factor 8 is activated by SENP1 deconjugating enzyme [37]. Because the KR mutant preserved Kctd15 functions we wondered if deSUMOylation might control the biological activity of Kctd15 such that SUMOylated Kctd15 represents the inactive form. To test this hypothesis we conjugated SUMO1 to the C–terminal end of Kctd15 (Kctd15-SUMO1, Figure 5A). This approach has been used, for example, for recombinant expression of proteins that are difficult to produce [38]. We tested this fusion construct in the assays applied before. Kctd15-SUMO1 was distributed in the cytosol and the nuclear fraction as its WT counterpart (data not shown). However, Kctd15-SUMO1 fusion protein was less efficient in inhibiting the AP2-Luc reporter than the WT protein, even though both proteins were expressed at similar levels (Figure 5B). The same result was obtained with Kctd15-SUMO2 fusion protein (data not shown). When injected into zebrafish embryos, Kctd15-SUMO1 mRNA failed to efficiently inhibit NC development whereas WT Kctd15 and the K252R mutant did, as already shown above (Figure 5C).

## Discussion

Here we identify a SUMO Interacting Motif (SIM) in the Kctd15 molecule. We provide evidence that the site is a target for SUMOylation, but mutation of the acceptor lysine to arginine does not affect Kctd15 stability or nucleocytoplasmic localization. In addition, non-SUMOylated Kctd15 inhibits AP-2 activity as well as NC formation similar to WT protein, but a SUMO fusion of Kctd15 was less efficient in inhibiting AP2-Luc reporter activity and much less efficient in blocking NC development in the embryo.

Reversible SUMO modification regulates the functional properties of many proteins in different biological processes. SUMOylation occurs on lysine residues within a region having the consensus motif ψKxE [39]. We found that Kctd15 contains a SUMO Interacting Motif at the C-terminal end, which is conserved between species (Figure 1A). Using the SUMOplot program we obtained predictions of minor SUMOylation sites in addition to the major C-terminal site in the human and zebrafish proteins (Figure 1). Mutation of the acceptor lysine residue in the consensus motif demonstrates that K278 (human) or K252 (zebrafish) is the only substrate for SUMO addition (Figure 2). SUMOplot analysis of Kctd proteins show that only Kctd1 shares the conserved domain at the C-terminal with Kctd15. In addition, while some Kctd members harbor one or more variation of the consensus motif, no SUMOylation sites where detected in other family members (Table S1).

SUMOylation has multiple roles; it is involved in nucleocytoplasmic transport of proteins [40], protein stability [27], DNA repair [41], chromosome packing [5], [42], but a function in transcriptional regulation stands out. Many reports highlight the ability of SUMO conjugation to turn on or increase the co-repressor potential of several co-regulators, whereas a few cases have been described where SUMO attachment stimulates transcriptional activity [3]. Studies from Eloranta and Hurst described transcriptional suppression of AP-2α, AP-2β and AP-2γ activity by interaction with UBC9 conjugating enzyme and consequent SUMO attachment [24]. In addition, previous results have shown that Kctd15 and Kctd1 are involved in downregulation of AP-2 activity [19], [43]. Because the transactivation potential of AP-2 is modulated through SUMO conjugation, and the AP-2 inhibitors Kctd15 and Kctd1 possess an identical SUMOylation domain, we considered the possibility that regulation of AP-2 may require SUMO conjugation of these two negative regulators. However, we found that SUMOylation is not required for the inhibition of the transcriptional activity of AP-2 (Figure 4).

Our observation that a Kctd15-SUMO fusion protein had greatly reduced inhibitory activity suggests that, for both reporter activation by AP-2 and NC development, it is the non-SUMOylated form of Kctd15 that has biological activity. This result implicates SUMOylation as a regulatory mechanism limiting the function of Kctd15. Thus we hypothesize that during normal NC development a pool of SUMOylated Kctd15 is expressed at the medial edge of the neural plate border overlapping with AP-2α (19), but failing to perturb NC formation because SUMOylation blocks its activity. In the rest of the neural plate border, Kctd15 would largely be represented by the deSUMOylated form, allowing it to counteract encroachment by the NC domain. In this way, regulation of SUMOylation status of Kctd15 would help to sharpen the definition of the NC territory.

Although the role of SUMOylation in nuclear targeting has been established for some proteins [44], the nuclear localization of many other proteins is unaffected by SUMOylation [45], [46]. Our data show that nuclear targeting of Kctd15 in HEK293T cells does not depend on the presence of an intact SUMOylation site, indicating that for Kctd15 SUMO modification is dispensable for nuclear transport (Figure 3D).

In addition, it has been demonstrated that there are extended motifs in the substrates that allow additional levels of control during SUMO conjugation [2]. Among them are the phosphorylation-dependent SUMOylation motif “PDSM” represented by ψKxExxSP [47], [48], and negatively charged residues within a 10 amino acid region downstream of the SIM, such as ψKxExxD/E, known as negatively charged aminoacid-dependent SUMOylation motif (NDSM) [11]. In both cases, a SUMOylation/acetylation switch (SAS) is believed to modulate the activity of the modified targets. Therefore, while SUMO attachment represses transcription, its replacement by acetyl groups will favor transcriptional activation [2]. Another variation in the modulation of SUMO activity is a phosphorylation-independent deacetylation-SUMOylation switch contained in the ψKxEP motif, where the proline is a target of acetylation [49], [50]. Notably, Kctd15 contains a proline at position +1 downstream of its SIM (Figure 1B). Thus it is possible that SAS occurs in Kctd15, with biological consequences that have yet to be explored.

Both AP-2 and KCDT15 may have a role in obesity and diabetes. Experimental evidence is available for the regulatory role of AP-2 in the expression of adipocyte differentiation and insulin signal transduction genes [51], [52], [53]. AP-2 acts as a repressor in this context, and while SUMOylation is involved in AP-2 repressor activity [24] it has not been studied in this context. As to KCTD15, genome wide association studies (GWAS) have indicated a connection to obesity [54]. A recent review [55] suggests that KCTD15 affects obesity through regulation of AP-2, mediated by SUMOylation. The authors propose that UBC9, the SUMO conjugating enzyme, SUMOylates KCTD15, and KCTD15-SUMO acts as connector between UBC9 and AP-2 to mediate AP-2 SUMOylation. Specifically, SUMOylation of KCTD15 would facilitate SUMO conjugation to lysine 10 (K10) in the AP-2 activation domain to repress AP-2 function [24], [55]. Our work raises two questions regarding this model; (1) as shown in this report, SUMOylation of KCTD15 is not required for its inhibition of AP-2 activity. (2) AP-2α has multiple isoforms [56]. The longer isoform, 1a, includes K10, but shorter isoforms such as 1b do not [56]. We showed previously that KCTD15 blocks the activity of AP-2α1a and 1b, as well as AP-2β and AP-2γ with high efficiency [19]. These observations appear to argue against the hypothesis of Williams et al., with the proviso that in both cases the context of inhibition was reporter activation and NC formation rather than regulation of obesity-related genes; thus the model could hold in the obesity context. Nevertheless, our observations that KCTD15 inhibition of AP-2 is based on binding to the activation domain with a specific requirement for proline 59 [19], together with the present finding that SUMOylation is not required for inhibition, raise questions about the model that will only be resolved by experimental exploration.

## Materials and Methods

This research has been approved by the *Eunice Kennedy Shriver* National Institute of Child Health and Human Development, Bethesda, MD, USA Animal Care and Use Committee.

### cDNAs and Plasmids

The Kctd15 and AP-2 plasmids have been described [16], [19]. SUMO1-T7 and V5-SUMO3 constructs were generously provided by Keiko Ozato.

### Cell culture and Transfection

HEK293T cells were grown in Dulbecco's Modified Eagle's medium supplemented with 10% fetal bovine serum. HEK293T cells were transfected using Xtreme-Gene HP (Roche).

### SUMOylation Assay

Cells were lysed in cold lysis buffer (0.5% Triton X-100, 50 mM Tris-HCl, pH 7.5, 150 mM NaCl, 5 mM EDTA) supplemented with complete Mini protease inhibitor cocktail (Roche) and 200 µM iodoacetamide (IAA) and 100 µg/ml of N-ethylmaleimide (NEM) to avoid SUMO detachment from target protein. Whole cell extracts were used in pull down and immunoblotting experiments [19].

Another approach to preserve SUMO conjugation of proteins was lysing the cells in SDS buffer [24]. HEK293T cells overexpressing the proteins of interest were kept on ice, washed once with cold PBS, 200 µl of SDS loading buffer preheated to 85°C was added to the wells, and the samples were heated at 95°C for 5 min. DNA was sheered using the Bioruptor Next Gen (Diagenode) before proteins were analyzed using the NuPAGE Bis-Tris Gel system 4–12% (Invitrogen). SuperSignal West Pico or Dura Chemiluminescent Substrates from Thermo Scientific were used for detection of horseradish peroxidase-conjugated antibodies.

### Subcellular Fractionation

This was performed using NE-PER Nuclear and Cytoplasmic Extraction Reagents (Thermo Scientific) following manufacturer's instructions.

### Antibodies

Anti-FLAG clone M2 antibody was from SIGMA, and anti-T7 Tag Monoclonal antibody was from Calbiochem (#69522-4). Human KCTD15 was detected with anti-KCTD15 MaxPab mouse polyclonal antibody (H00079047-B01P, Abnova), or rabbit anti-human KCTD15 polyclonal antibody (LS C110024/25441, LifeSpan Biosciences). The latter was also used to detect zebrafish Kctd15. Anti-α-Tubulin antibody (Calbiochem) and anti-PARP (Cell Signaling) were used as control. HRP-conjugated secondary antibodies were from Jackson.

### mRNA Microinjection

mRNAs were synthesized from linearized constructs using the mMESSAGE mMACHINE kit (Ambion Inc.) according to the manufacturer's instruction. RNA quality was checked on formaldehyde gels. RNA or plasmids were injected into the yolk or the cell of 1-cell stage embryos. Zebrafish embryos were collected at bud or 1–3 somite stage for in situ hybridization (ISH).

### Whole mount in situ hybridization

The *foxD3* probe has been described [16]. Antisense riboprobes were generated following manufacturers' instructions (Roche). Whole mount ISH was performed as described [57].

### Luciferase assay

pGL3-AP2-Luc reporter was used to study AP-2 mutant activity [19], [56]. HEK293T cells (24 hr after transfection) were lysed in 1× Passive Buffer (Promega). Luciferase assay was performed using Dual Luciferase Reporter Assay System from Promega. Each luciferase activity was measured at least three times.

## Supporting Information

Table S1 **SUMOplot analysis of Kctd family members.** The SUMOplot Analysis Program predicts and scores SUMO interacting motifs in proteins of interest. We tested all members of the human KCTD family in the software, including the newly identified family member (see ref. 15 in main text). The numbers in red indicate the sites with high probability for SUMO attachment. No., number; Pos., position.
(PDF)
