# Supplementary material for: The BTB-Containing Protein Kctd15 Is SUMOylated In Vivo
Source: PLoS One. 2013 Sep 24;8(9):e75016. doi: 10.1371/journal.pone.0075016 (PMC3782465; doi:10.1371/journal.pone.0075016)
Supplement: Table S1 — SUMOplot analysis of Kctd family members. The SUMOplot Analysis Program predicts and scores SUMO interacting motifs in proteins of interest. We tested all members of the human KCTD family in the software, including the newly identified family member (see ref. 15 in main text). The numbers in red indicate the sites with high probability for SUMO attachment. No., number; Pos., position. (PDF) [file pone.0075016.s001.pdf]

Table S1. *SUMOplot analysis for Kctd family members.*

	No.	Pos.	Group	Score
KCTD1	1	K860	PSVIR **IKQE** PLD	0.94
	2	K527	QVGPD **VKSE** AAPKR	0.93
	3	K572	VVVVS **VKHD** PLPLL	0.93
	4	K736	LEMER **WKQD** RETGR	0.64
	5	K721	LLYEE **AKYF** QLQPM	0.44
	6	K269	LAAVI **RKLE** EQGAG	0.44
KCTD2	1	K209	TMSDG **WKFE** QLISI	0.64
	2	K113	PELDS **DKDE** TGAYL	0.50
KCTD3	1	K299	ATSHT **GKVG** VWNAV	0.50
	2	K805	PSPRH **KKSD** SSGQE	0.48
	3	K344	YYIDM **QKFP** LRMKD	0.39
	4	K769	GFLGR **KKVP** YLASS	0.37
KCTD4	1	K201	QFKYF **IKSE** NGTRL	0.94
	2	K211	GTRLV **LKED** NTFVC	0.91
	3	K224	CTLET **LKFE** AIMMA	0.91
	4	K233	AIMMA **LKCG** FRLLT	0.73
	5	K197	SNIIQ **FKYF** IKSEN	0.50
KCTD5	1	K180	TMSDG **WKFE** QLVSI	0.64
	2	K84	PDLDS **DKDE** TGAYL	0.50
	3	K67	TLCRD **PKSF** LYRLC	0.26
KCTD6	1	K84	TLPLD **FKEF** DLLRK	0.50
	2	K234	FCRLA **RKTD** D	0.44
KCTD7	1	K148	ENMQP **LKGE** KVRQA	0.91
	2	K194	LKVCV **FKEE** MPITP	0.85
KCTD8	1	K330	QKIVS **PKQE** HEDRK	0.61
	2	K337	QEHED **RKHD** KVTDK	0.44
	3	K395	LDRPS **KKAP** VQWIP	0.37
	4	K470	QSELL **QKYG** L	0.33
KCTD9	1	K46	TSVYN **GKGG** LIDDI	0.50
	2	K13	FLNGS **PKNG** KVVAV	0.43
	3	K203	HSPIS **RKEF** VRFLL	0.09
KCTD10	1	K303	VRRIH **IKRP** DDRAH	0.84
	2	K82	LIDRC **GKHF** GTILN	0.32
KCTD11	1	K31	FIDRD **GKAF** RHILN	0.32
KCTD12	1	K148	SRRGV **HKEG** SLGDE	0.34
KCTD13	1	K140	QLALQ **QKRE** TLSPL	0.50
KCTD14	1	K200	MFKSV **VKFG** PWKAV	0.76
	2	K53	TLGTL **RKFP** GSKLA	0.34
	3	K193	CFLQD **KKMF** KSVVK	0.13
	4	K136	GEQVS **RKQF** LLQVP	0.09
KCTD15	1	K278	PTAVR **IKQE** PLD	0.94
	2	K129	LLPDD **FKDF** SLLYE	0.50
KCTD16	1	K107	PEKGR **LKRE** AEYFQ	0.91
	2	K406	QDFLK **IKIP** DRFPE	0.84
	3	K347	TLDRP **IKKG** PVQLI	0.77
	4	K296	CCCKN **GKGD** KEGES	0.67
	5	K58	WKMFS **PKRD** TANDL	0.61
	6	K362	QSEMR **RKSD** LLRTL	0.44
KCTD17	1	K54	TLCRE **QKSF** LSRLC	0.15
KCTD18	1	K49	SGRFP **LKTD** ESGAC	0.91
	2	K188	HSKGI **FKRE** AGNNV	0.85
	3	K5	MEG **HKAE** EEVLD	0.52
	4	K369	KVLLS **DKKP** TPQRV	0.39
KCTD19	1	K875	LAITG **FKDD** RHTQE	0.85
	2	K145	PSEFP **IKSP** AFTGL	0.84
	3	K296	ALGLL **VKYP** DSALG	0.82
	4	K416	TLQTL **LKYP** ELLSN	0.80
	5	K106	QTLDN **LKEG** KHHLR	0.73
	6	K184	PLDLV **AKYP** SLVTE	0.69
	7	K355	LCAFL **DKRD** ITYEP	0.50
	8	K621	TINLT **QKSE** TKDPP	0.50
	9	K155	FTGLH **DKAP** LGLMD	0.39
	10	K695	HSTAS **EKDP** GPQAG	0.39
	11	K710	GAGAK **DKGP** EPTFK	0.39
	12	K838	MDSIR **QKDP** KAITA	0.39
	13	K456	NFLRL **GKLF** LPSEF	0.32
	14	K897	TLPFA **RKYG** RCMDL	0.27
	15	K862	TLHIS **PKQF** VVDLL	0.26
KCTD20	1	K252	IMVGC **AKKG** ERECH	0.62
	2	K161	FTRPN **EKGE** YEIAE	0.50
	3	K357	IQMSW **EKEE** GKSRH	0.50
	4	K253	MVGCA **KKGE** RECHI	0.48
	5	K233	SNDGA **HKQF** DHYLE	0.17
KCTD21	1	K36	GAMFS **GKMP** TKRDS	0.57
	2	K54	FIDRD **GKVF** RYILN	0.32
SHKBP1	1	K44	GRIST **LKDE** TGAIF	0.91
	2	K576	AMEIA **GKVD** IKALG	0.67
	3	K297	ATSHT **GKVG** VWNAV	0.50
	4	K342	YYIDV **QKFP** LRMKD	0.39
	5	K15	HLNVG **GKRF** STSRQ	0.32
Btbd10	1	K41	TSSRI **AKGG** VDHTK	0.62
	2	K157	FVYEN **AKEG** ARNIR	0.62
	3	K211	FTRPN **EKGE** YEVAE	0.50
	4	K407	IRMSW **EKEE** GKSRH	0.50
	5	K383	YKEKV **KKRP** GGRPE	0.37
TNFAIP1	1	K144	PIITS **LKEE** ERLIE	0.91
	2	K21	PKLSG **FKGG** GLGNK	0.68
	3	K127	QSALQ **DKKD** SYQPV	0.50
	4	K67	MEVLT **DKEG** WILID	0.33
	5	K78	LIDRC **GKHF** GTILN	0.32
KCNRG	1	K24	TRFST **IKQF** PASRL	0.59
	2	K14	TLNVG **GKIF** TTRFS	0.32
